# Supplementary figures and images for: Gene expression signatures as candidate biomarkers of response to PD-1 blockade in non-small cell lung cancers
Source: PLoS One. 2021 Nov 29;16(11):e0260500. doi: 10.1371/journal.pone.0260500 (PMC8629226; doi:10.1371/journal.pone.0260500)

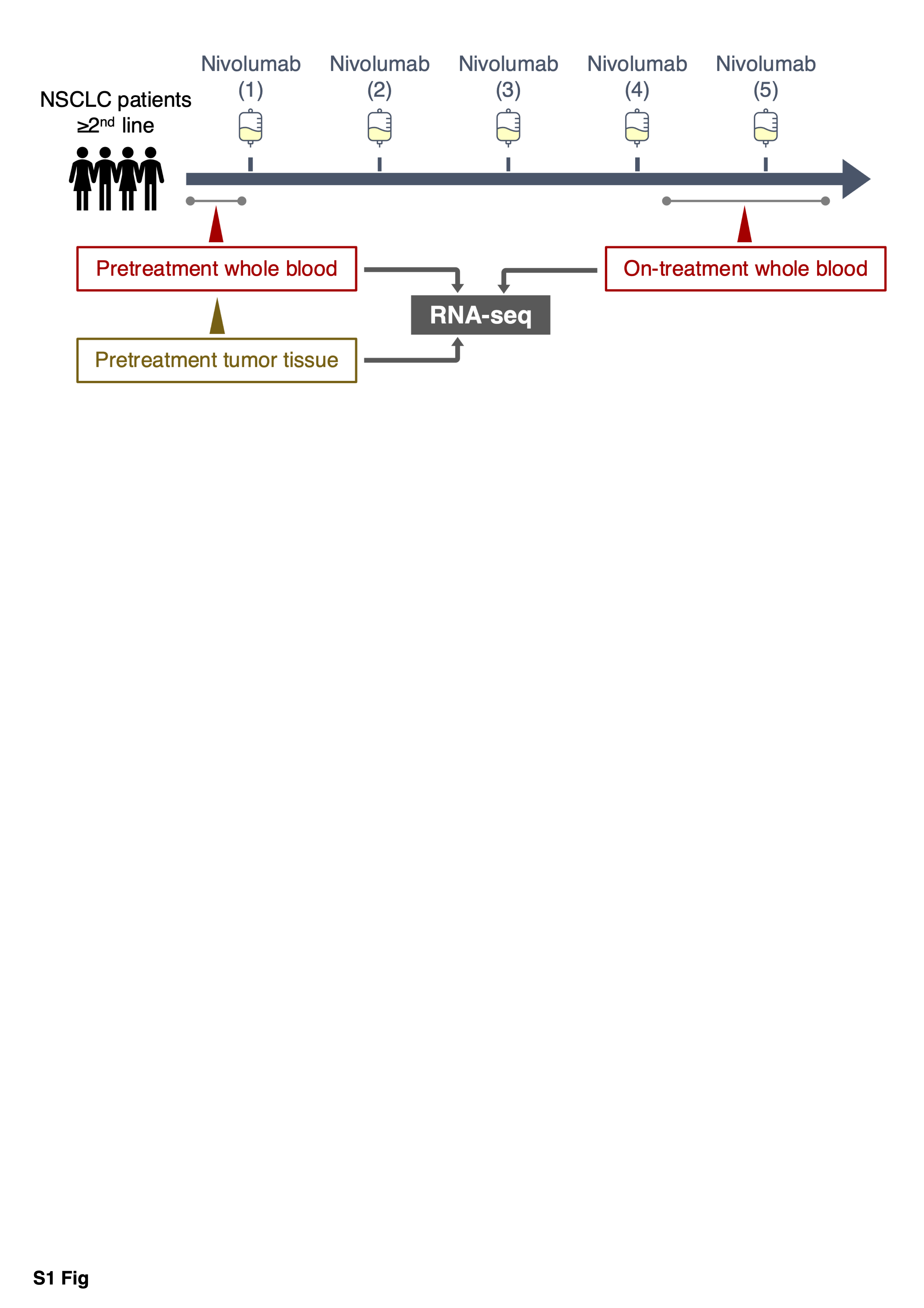

Supplement: S1 Fig — (TIFF) [file pone.0260500.s001.tiff]

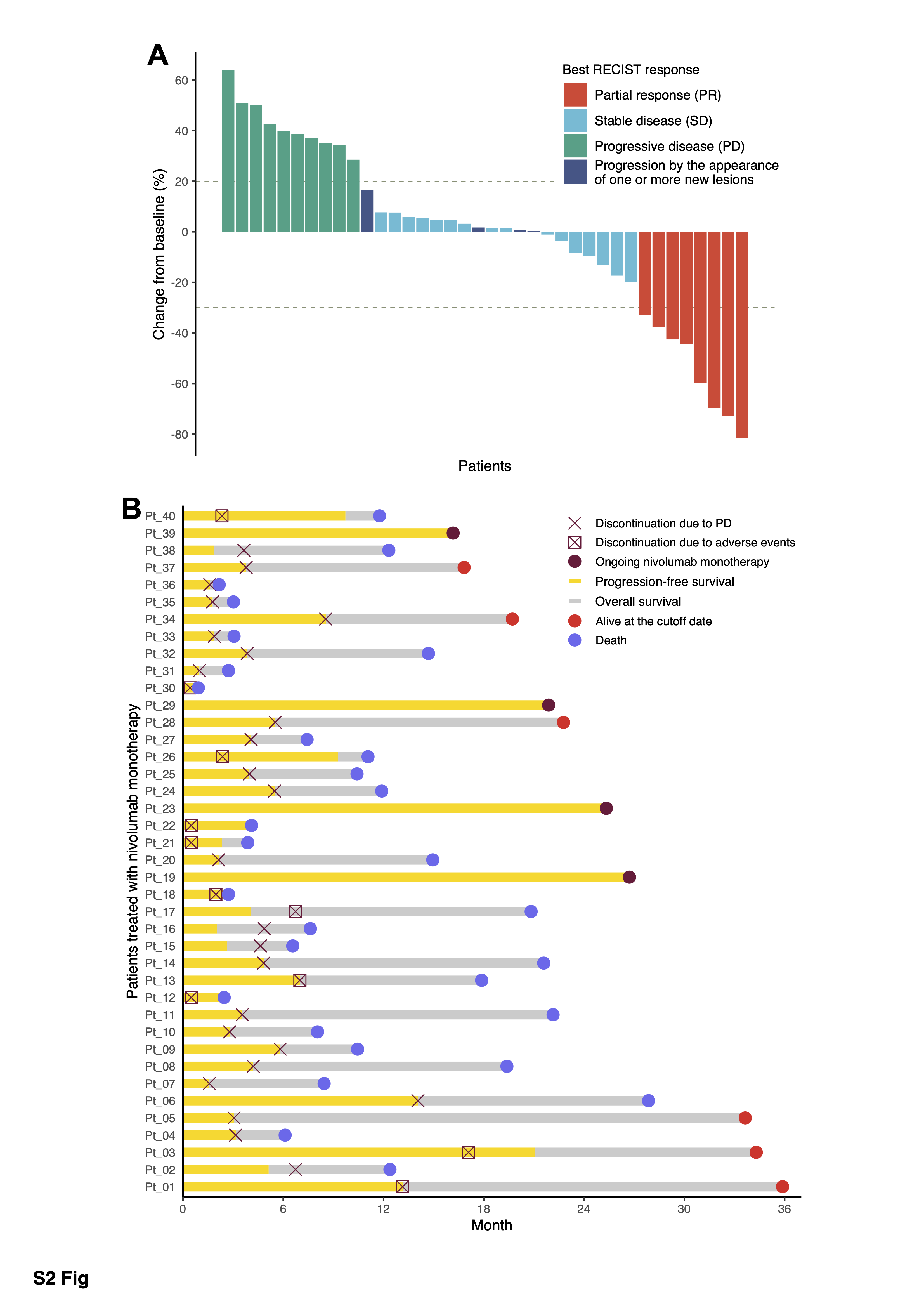

Supplement: S2 Fig — A, Waterfall plot of the best percentage change from baseline during nivolumab monotherapy according to RECIST v1.1. B, Swimmer plot of all 40 patients treated with nivolumab monotherapy. PD, progressive disease. (TIFF) [file pone.0260500.s002.tiff]

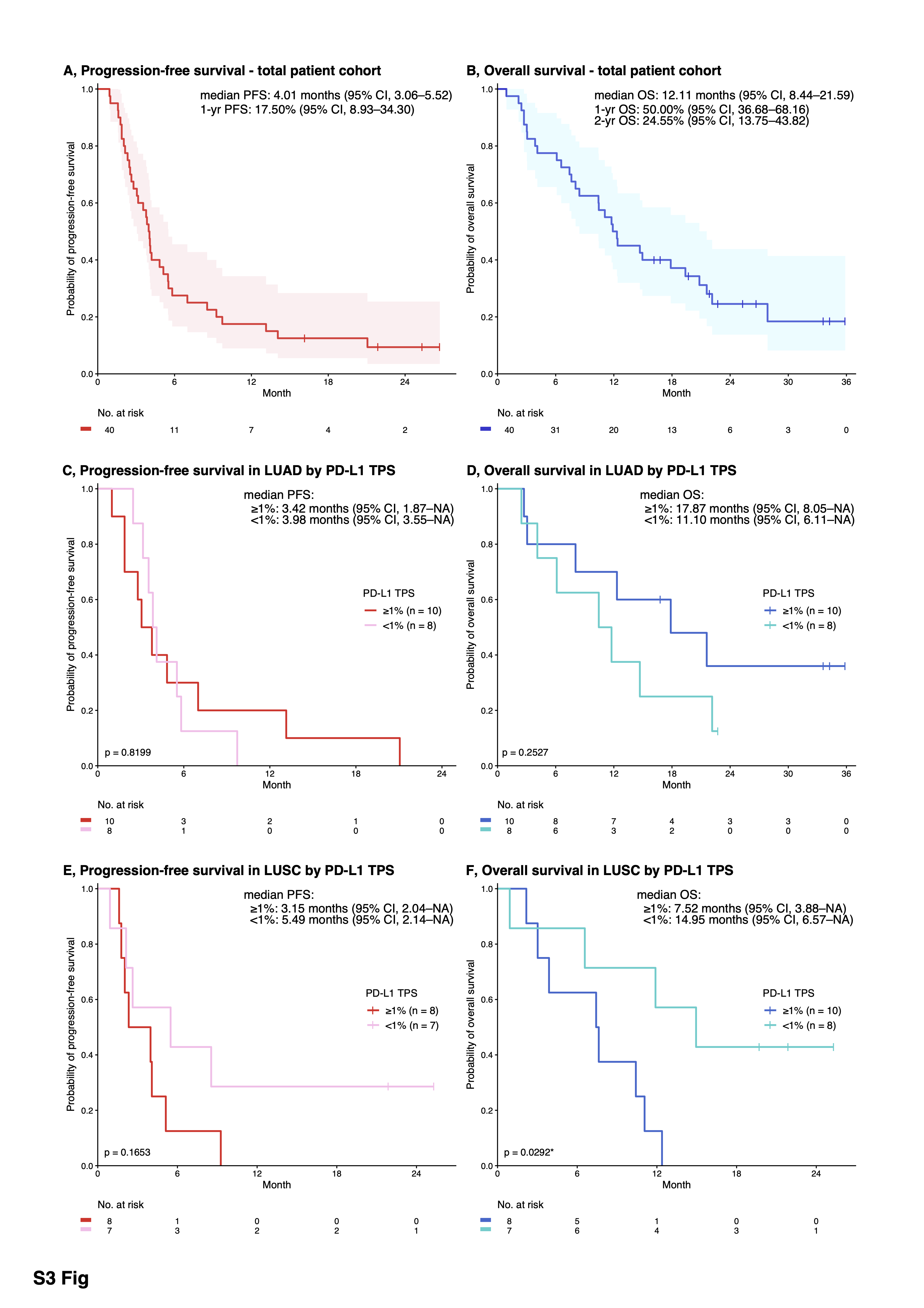

Supplement: S3 Fig — A–B, the total patient cohort and C–D, LUAD or E–F, LUSC patients with PD-L1 TPS ≥ 1% versus < 1%. The p-values were calculated by the two-sided log-rank test. (TIFF) [file pone.0260500.s003.tiff]

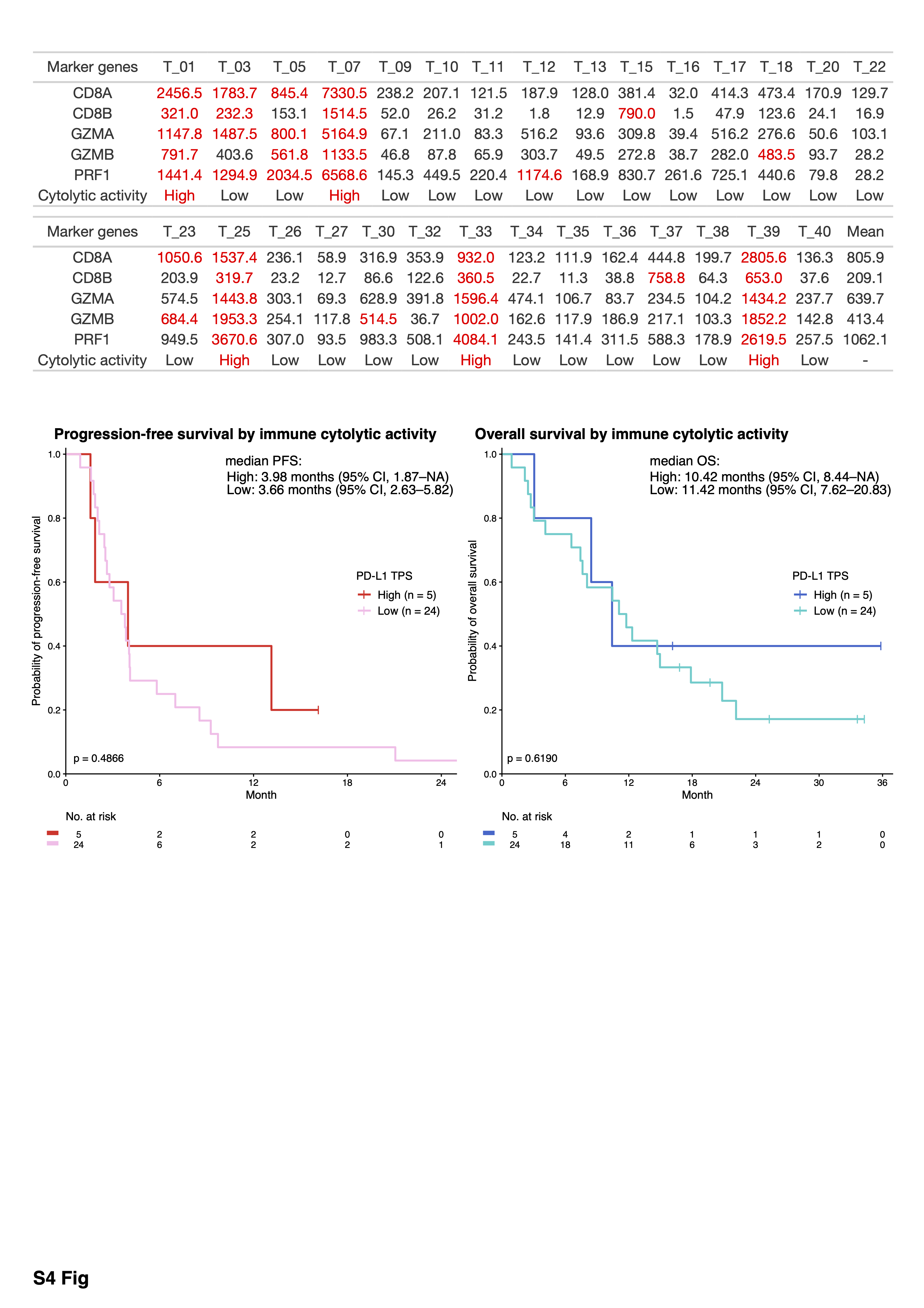

Supplement: S4 Fig — For each tumor sample, the immune cytolytic activity was estimated as the average expression level of the marker genes (CD8A, CD8B, GZMA, GZMB and PRF1). Patients with all expression levels above the average are defined as ‘High’; the others, as ‘Low’. In the upper panel, above-average values are shown in red. The lower panel illustrates Kaplan-Meier estimates of PFS and OS of patients stratified by the estimated immune cytolytic activity. The p-values were calculated by the two-sided log-rank test. (TIFF) [file pone.0260500.s004.tiff]

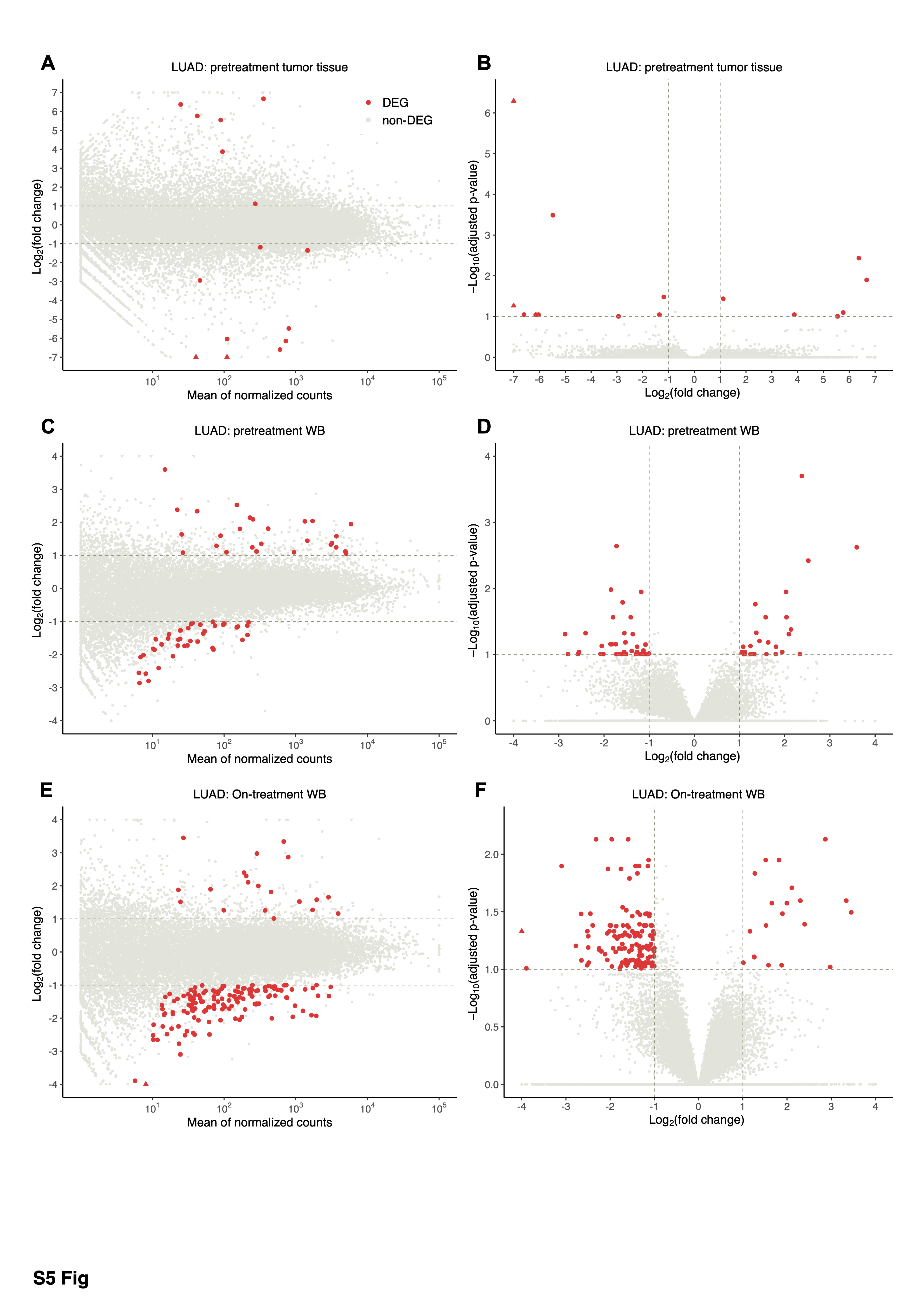

Supplement: S5 Fig — A–B, MA plot (A) and volcano plot (B) of DEGs in pretreatment tumor tissues. C–D, MA plot (C) and volcano plot (D) of DEGs in pretreatment WB. E–F, MA plot (E) and volcano plot (F) of DEGs in on-treatment WB. Red dots represent DEGs [adjusted p-value < 0.10 and |log2(fold change)| ≥ 1]. Triangles and diamonds represent genes with log2(fold change) and normalized counts, respectively, out of the plot scale. The horizontal lines in the MA plots and vertical lines in the volcano plots indicate the thresholds log2(fold change) = 1 or −1. The horizontal lines in the volcano plots indicate the threshold −log10(adjusted p-value) = 1. (TIFF) [file pone.0260500.s005.tiff]

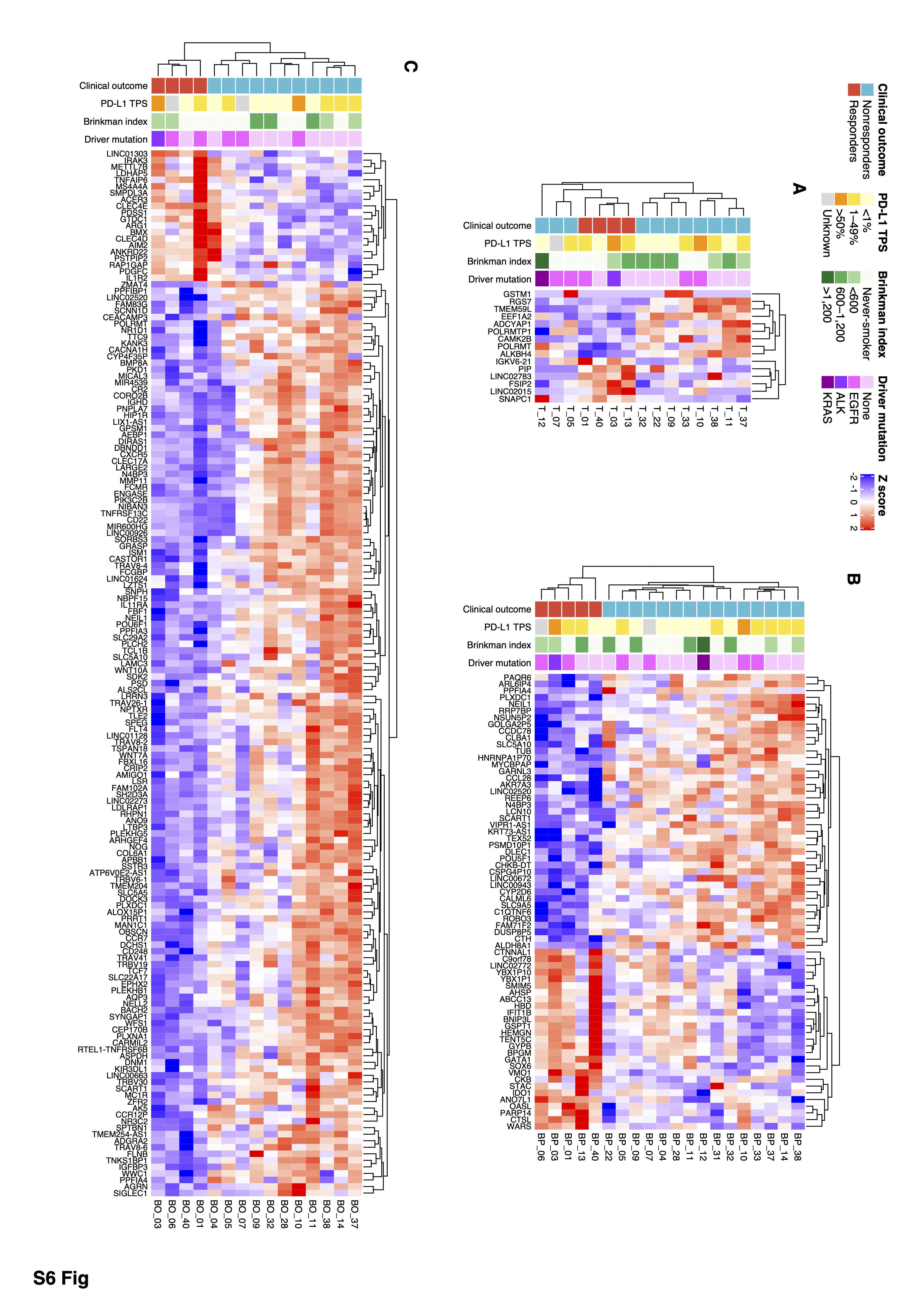

Supplement: S6 Fig — Heatmaps of DEGs between responders and nonresponders with hierarchical clustering of samples: A, from pretreatment tumor tissues (n = 15), B, pretreatment WB (n = 20), and C, on-treatment WB (n = 15). The DEGs clearly differentiated between responders and nonresponders in all three datasets. (TIFF) [file pone.0260500.s006.tiff]

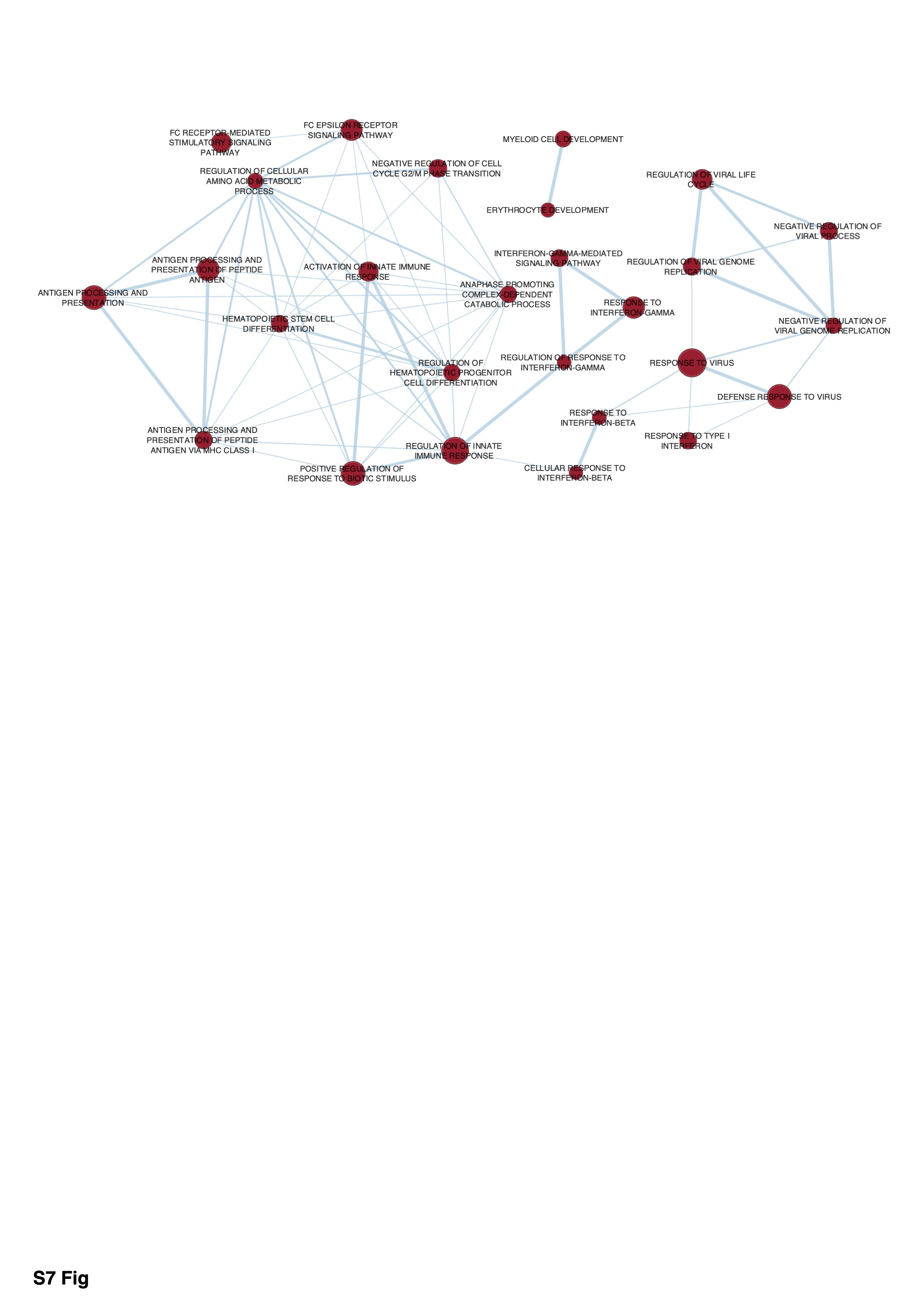

Supplement: S7 Fig — Each node denotes a distinct gene set, and the size of the node is proportional to the number of genes in the set. The thickness of the edges (pale blue lines) represents the degree of overlap between the two connected gene sets. (TIFF) [file pone.0260500.s007.tiff]

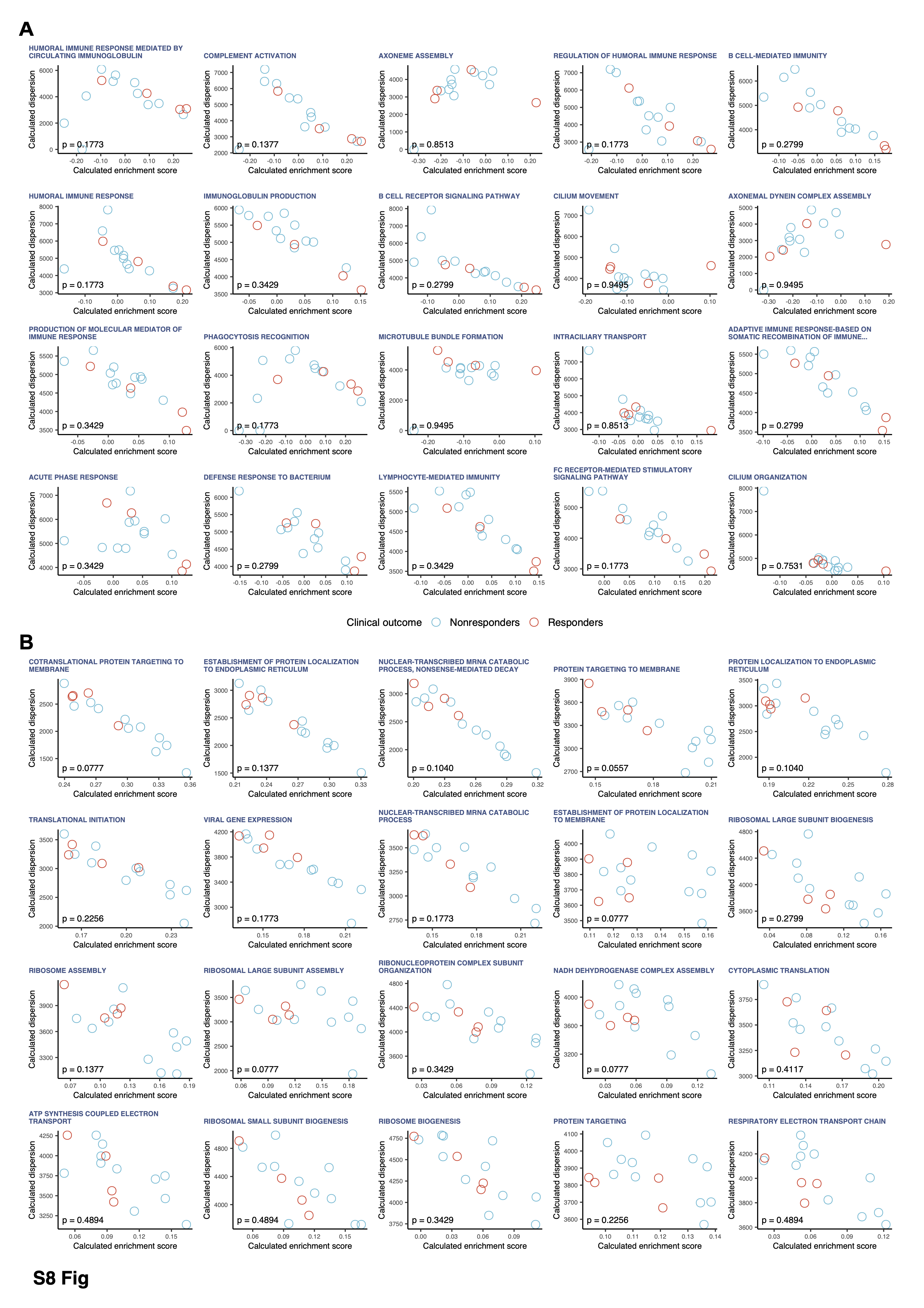

Supplement: S8 Fig — For each gene set in the top 20 GSEA gene sets that were enriched in pretreatment tumor tissues of responders (A) and nonresponders (B) with LUAD, the enrichment scores and dispersions were calculated using singscore. Red circles denote responders (n = 4); cyan circles, nonresponders (n = 11). The enrichment scores were analyzed using the Wilcoxon rank sum test. (TIFF) [file pone.0260500.s008.tiff]

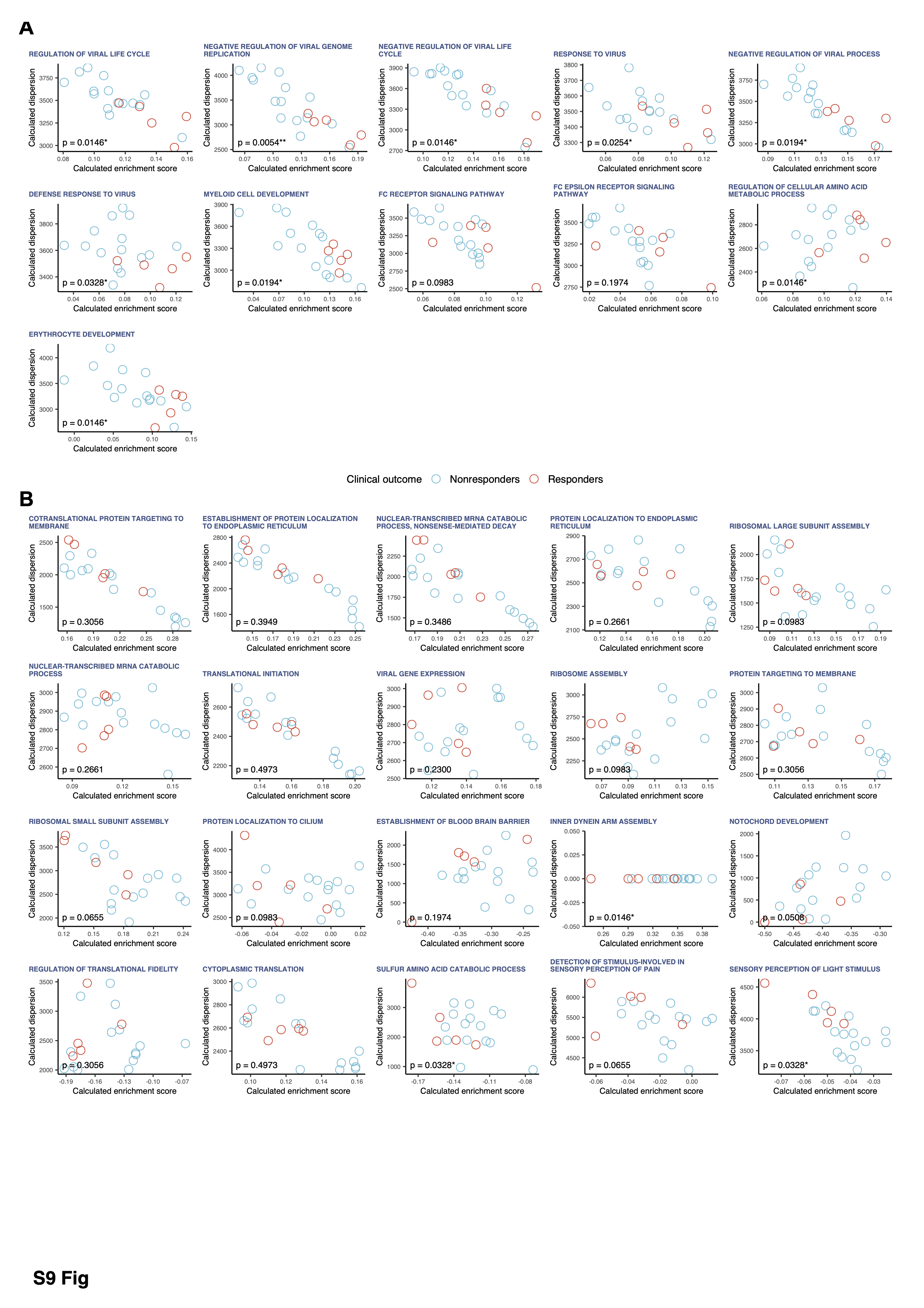

Supplement: S9 Fig — For each gene set in the top 20 GSEA gene sets that were enriched in pretreatment WB of responders (A, except for those shown in Fig 2A) and nonresponders (B) with LUAD, the enrichment scores and dispersions were calculated using singscore. Red circles denote responders (n = 5); cyan circles, nonresponders (n = 15). The enrichment scores were analyzed using the Wilcoxon rank sum test (*p < 0.05 and **p < 0.01). (TIFF) [file pone.0260500.s009.tiff]

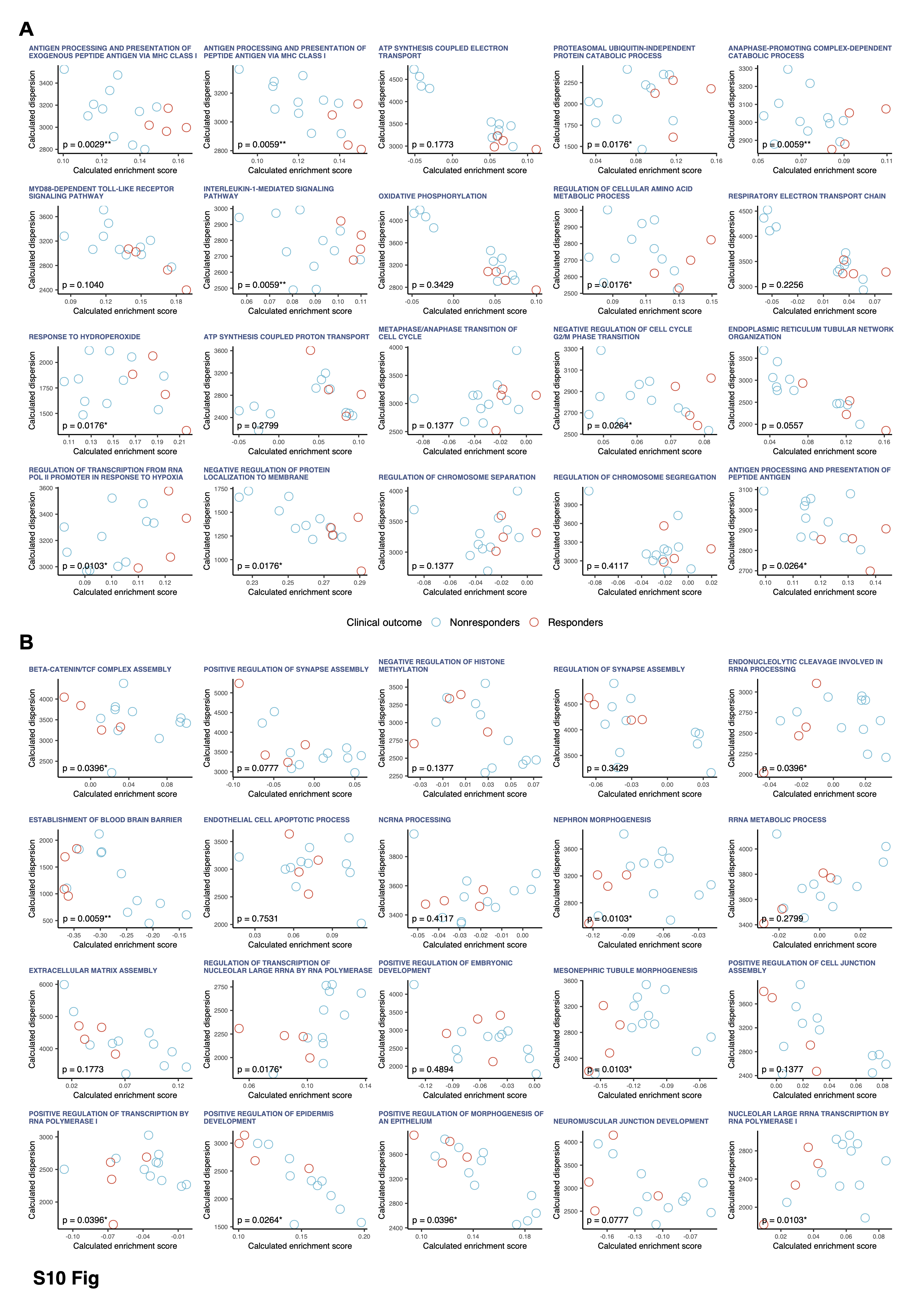

Supplement: S10 Fig — For each gene set in the top 20 GSEA gene sets that were enriched in on-treatment WB of responders (A) and nonresponders (B) with LUAD, the enrichment scores and dispersions were calculated using singscore. Red circles denote responders (n = 4); cyan circles, nonresponders (n = 11). The enrichment scores were analyzed using the Wilcoxon rank sum test (*p < 0.05 and **p < 0.01). (TIFF) [file pone.0260500.s010.tiff]

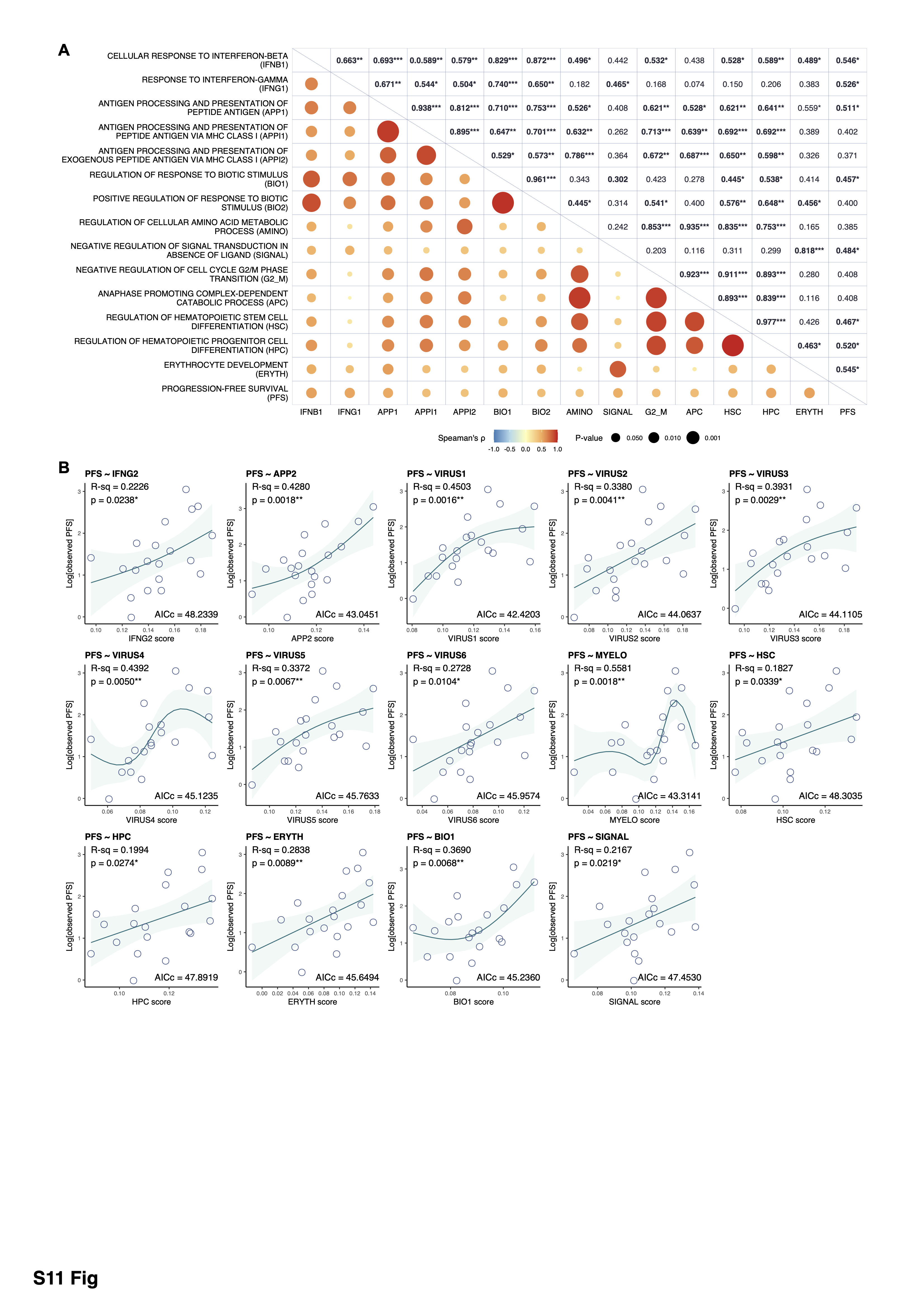

Supplement: S11 Fig — A, Spearman correlation matrix between PFS and the enrichment scores in pretreatment WB. The upper triangular region shows the values of the Spearman’s ρ correlation coefficients (significant correlations are in bold; *p < 0.05, **p < 0.01 and ***p < 0.001). In the lower triangular region, positive correlations are visualized in red and negative correlations in blue. The color intensity is proportional to the value of Spearman’s ρ and the size of the circle to the p-value. B, Scatter plots showing the relationships between PFS and the enrichment score of the above gene sets in pretreatment WB, with a fitted line representing the regression model using a cubic spline and 95% confidence interval. The accuracy of the fit was assessed by calculating the adjusted R-squared (R-sq) and p-values (*p < 0.05 and **p < 0.01). (TIFF) [file pone.0260500.s011.tiff]

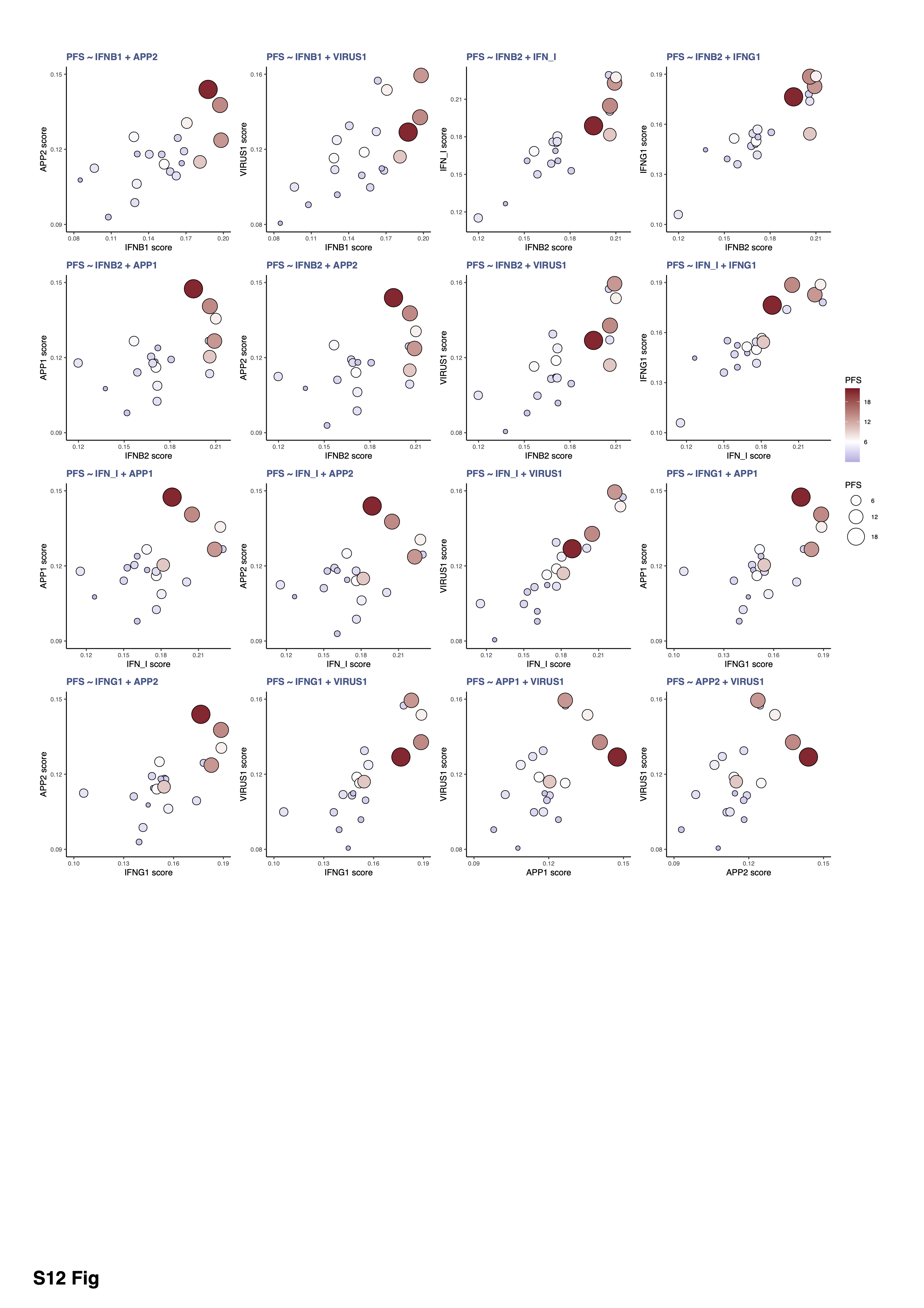

Supplement: S12 Fig — Each bubble represents a patient, and the size of the bubble is proportional to the PFS time. On a gradient color scale based on the PFS time, bubbles representing responders were assigned colors ranging from white to dark red; nonresponders, ranging from white to lavender. (TIFF) [file pone.0260500.s012.tiff]

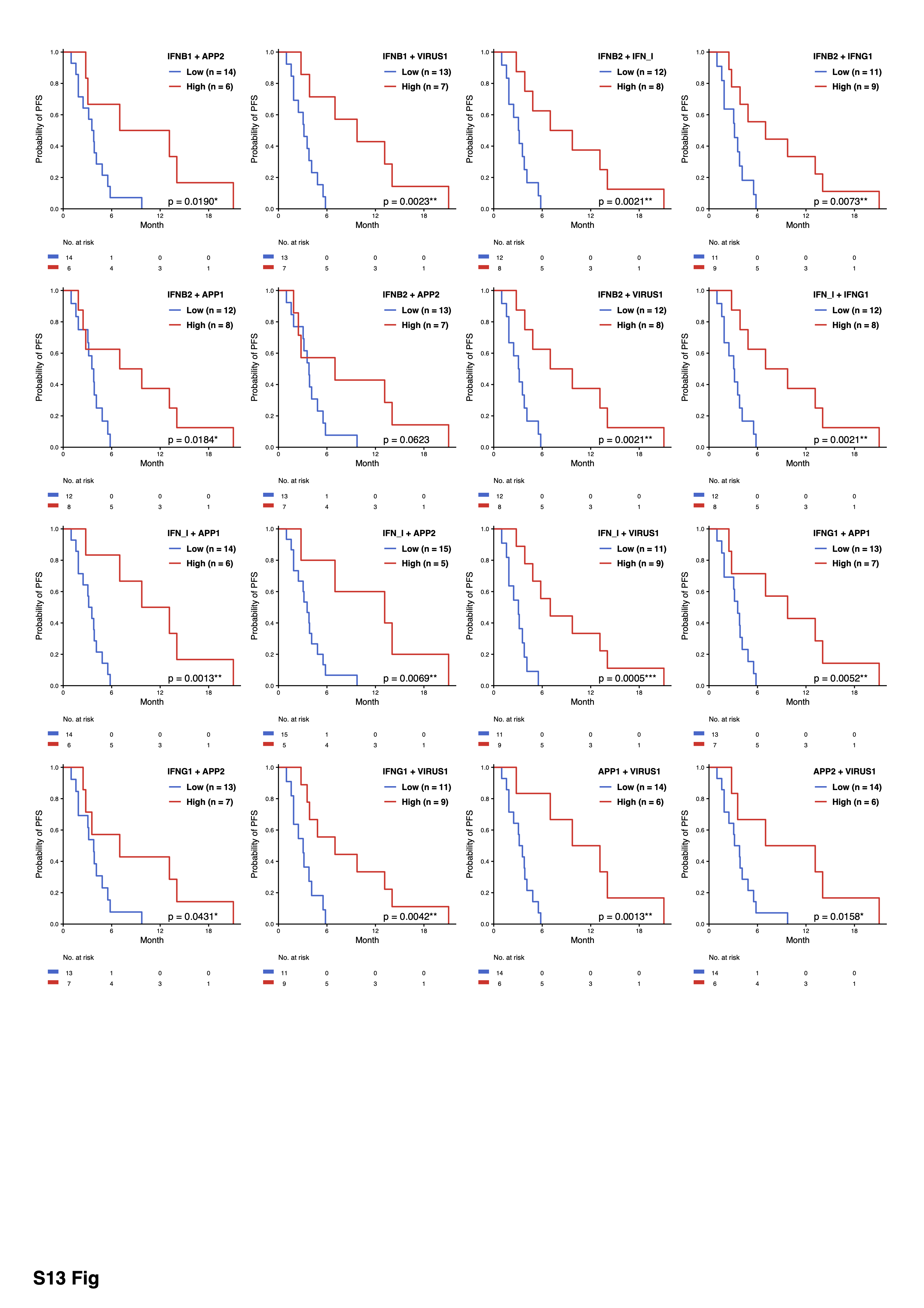

Supplement: S13 Fig — LUAD patients with both scores above the median are defined as ‘High’; the others, as ‘Low’. The p-values were calculated by the two-sided log-rank test (*p < 0.05, **p < 0.01, and ***p < 0.001). (TIFF) [file pone.0260500.s013.tiff]

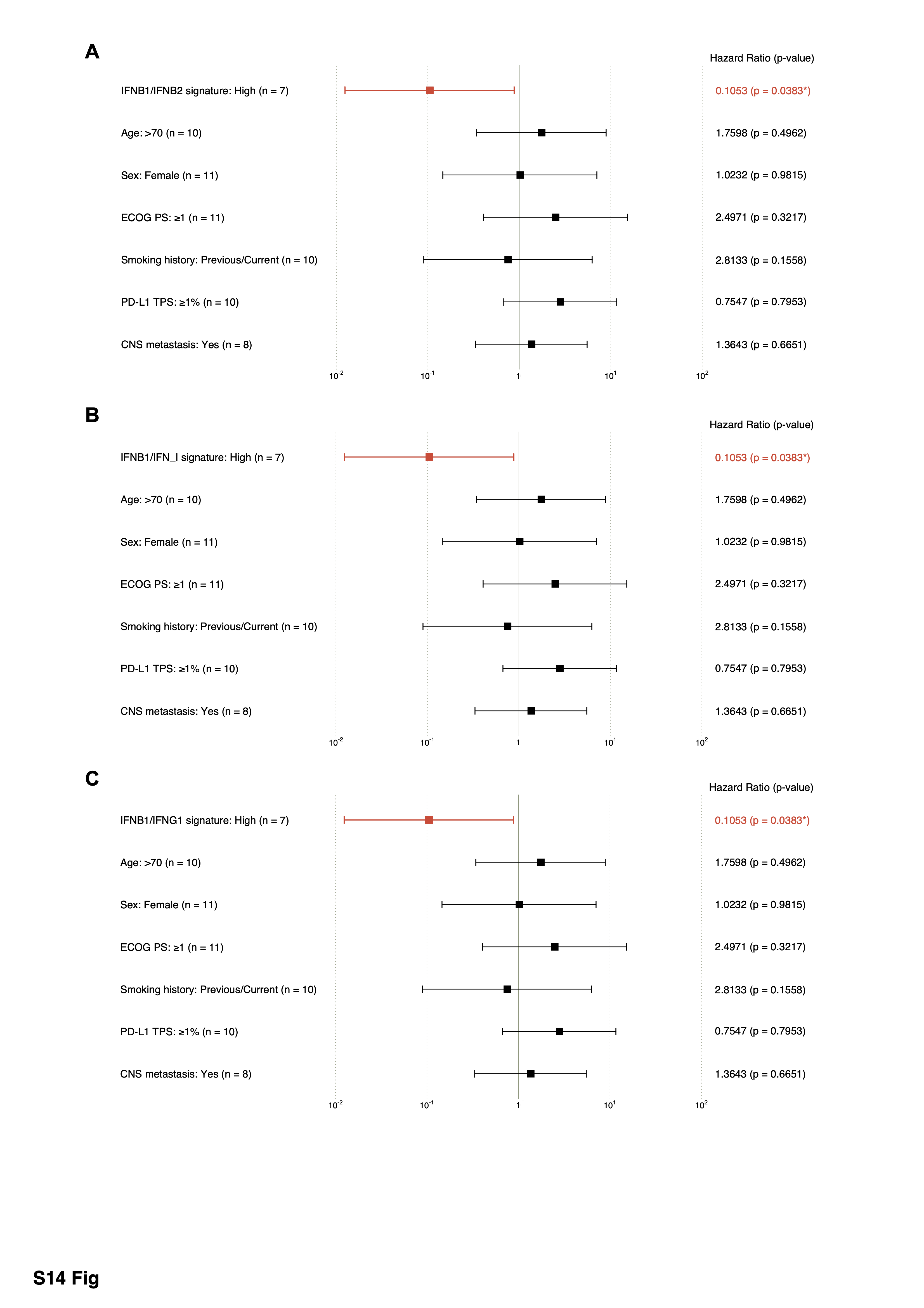

Supplement: S14 Fig — Squares represent estimated hazard ratios and whiskers represent the 95% confidence intervals. Hazard ratios less than 1 indicate improved PFS time (*p < 0.05). (TIFF) [file pone.0260500.s014.tiff]

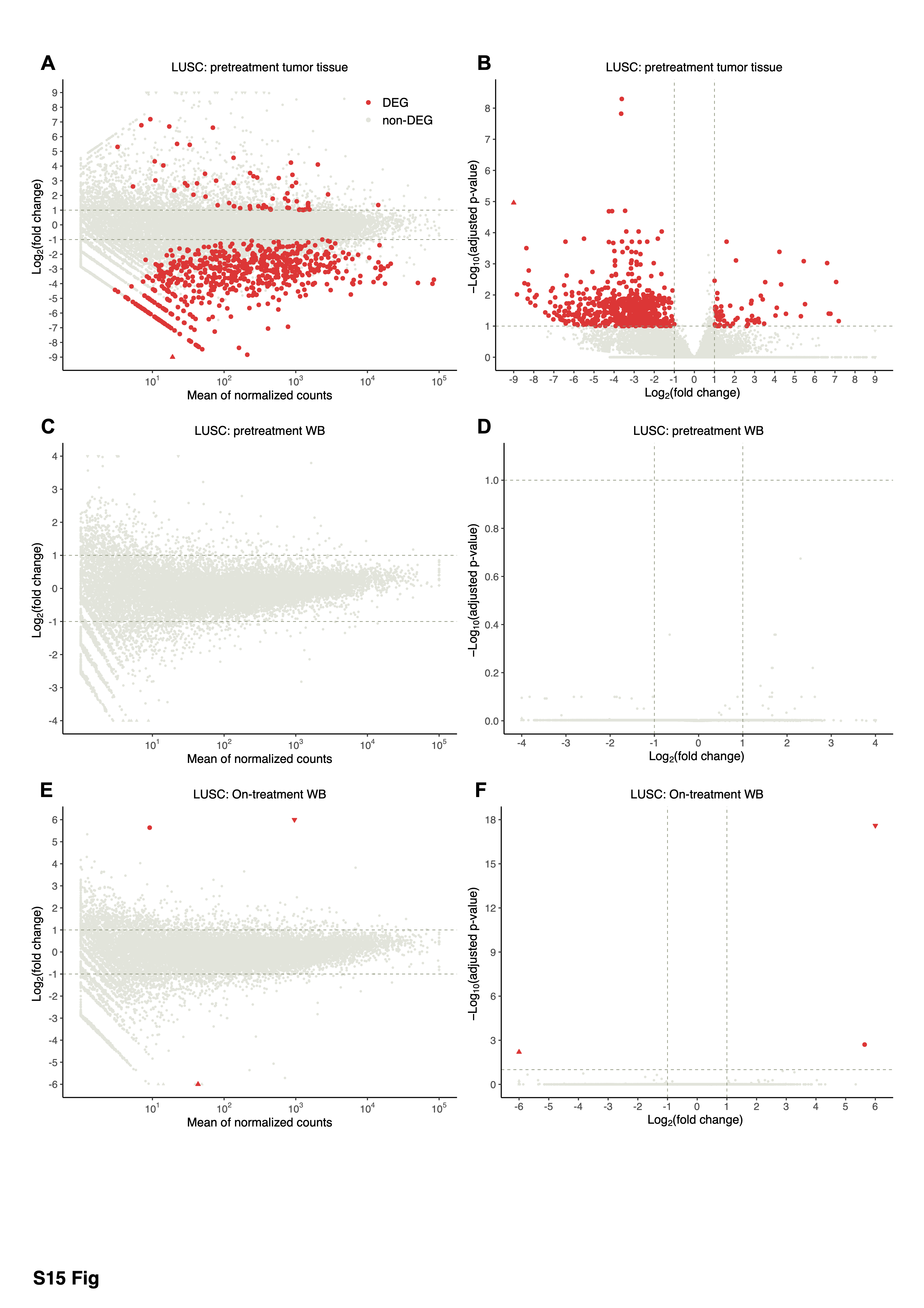

Supplement: S15 Fig — A–B, MA plot (A) and volcano plot (B) of DEGs in pretreatment tumor tissues. C–D, MA plot (C) and volcano plot (D) of DEGs in pretreatment WB. E–F, MA plot (E) and volcano plot (F) of DEGs in on-treatment WB. Red dots represent DEGs [adjusted p-value < 0.10 and |log2(fold change)| ≥ 1]. Triangles and diamonds represent genes with log2(fold change) and normalized counts, respectively, out of the plot scale. The horizontal lines in the MA plots and vertical lines in the volcano plots indicate the thresholds log2(fold change) = 1 or −1. The horizontal lines in the volcano plots indicate the threshold −log10(adjusted p-value) = 1. (TIFF) [file pone.0260500.s015.tiff]

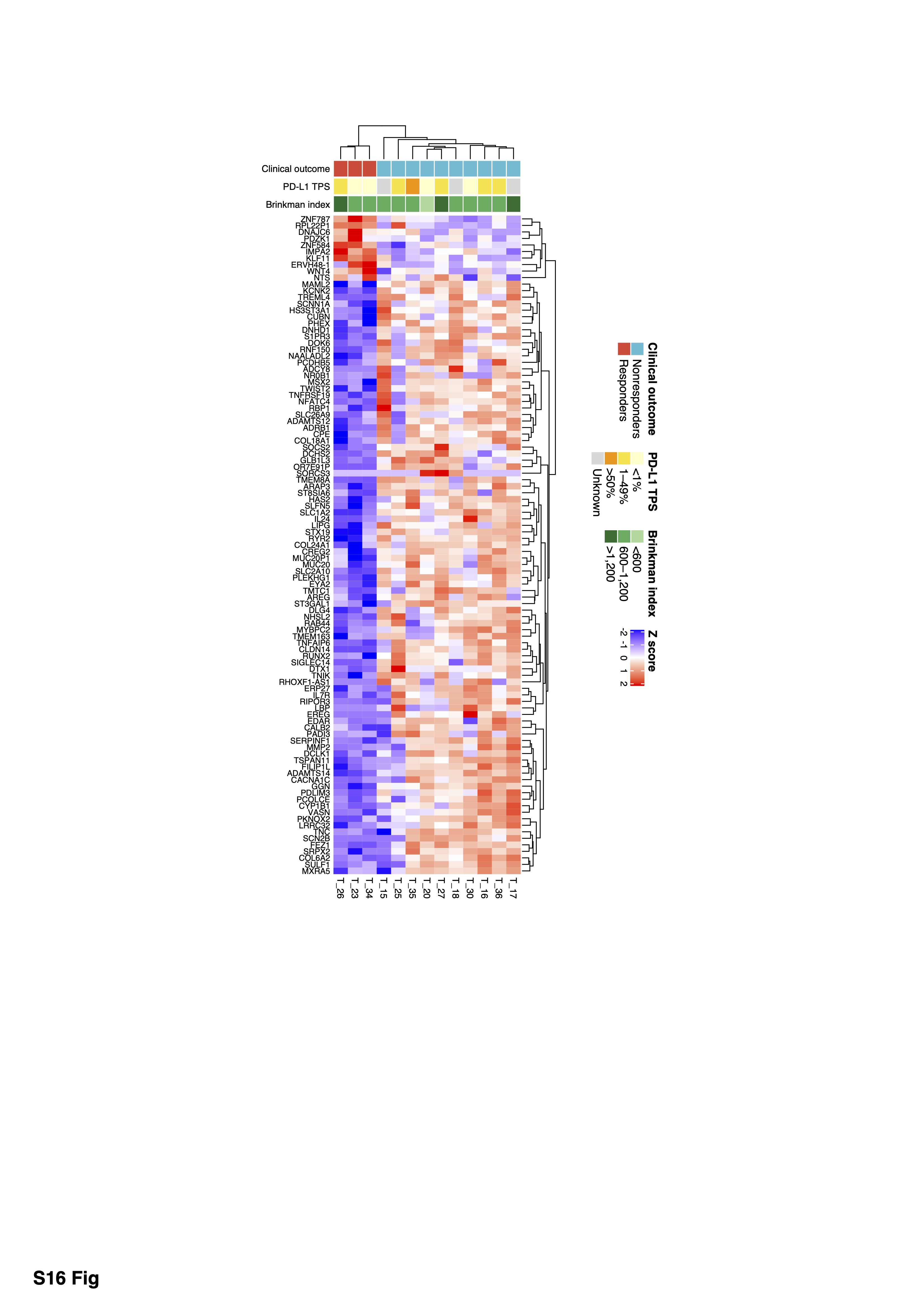

Supplement: S16 Fig — Heatmaps of DEGs between responders and nonresponders with hierarchical clustering of samples from pretreatment tumor tissues (n = 13). (TIFF) [file pone.0260500.s016.tiff]

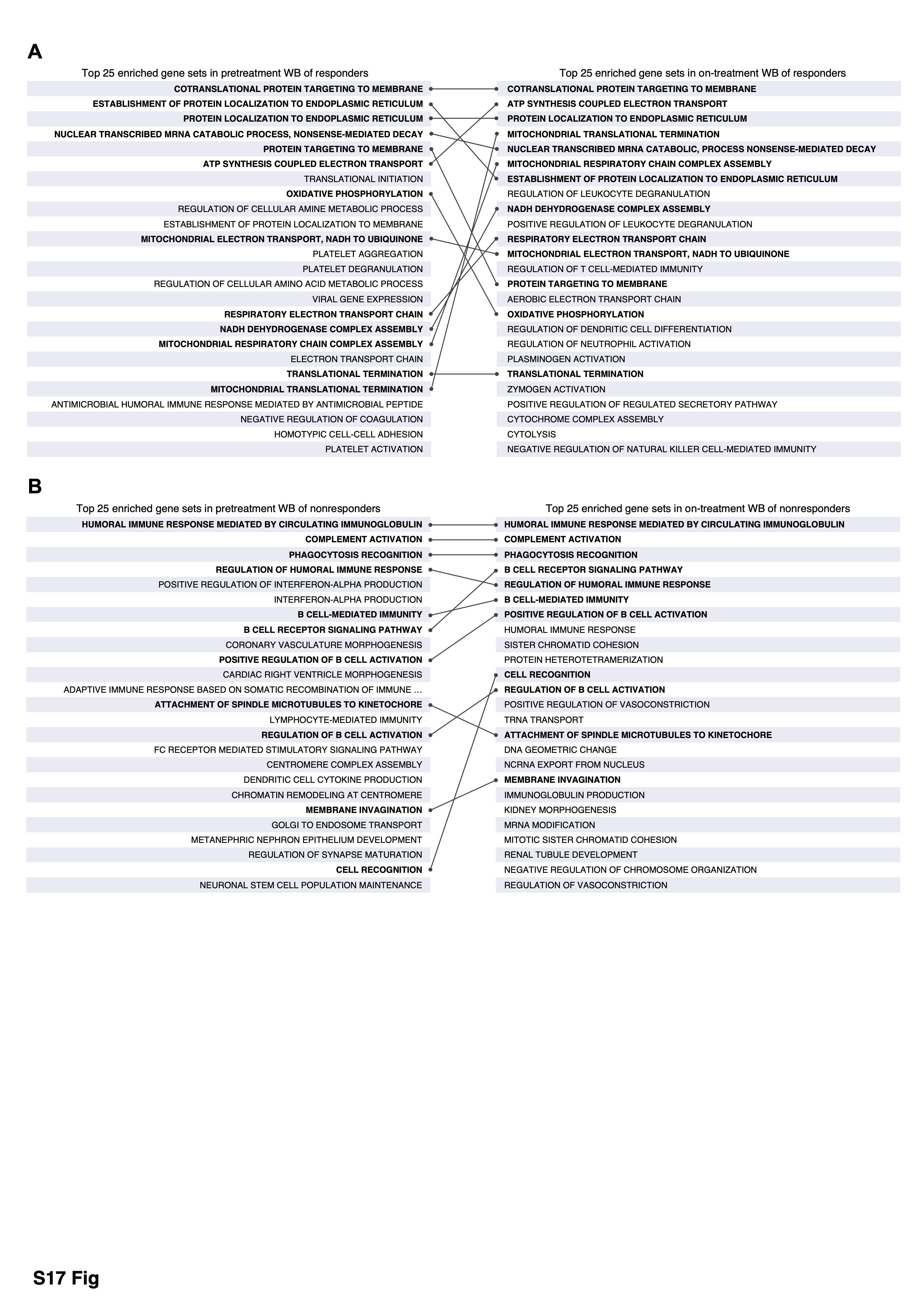

Supplement: S17 Fig — Top 25 enriched gene sets in responders (A) and in nonresponders (B). The left panel shows the list for pretreatment WB; the right panel, on-treatment WB. The enriched gene sets common between pretreatment and on-treatment WB are shown in bold and connected by solid lines. (TIFF) [file pone.0260500.s017.tiff]

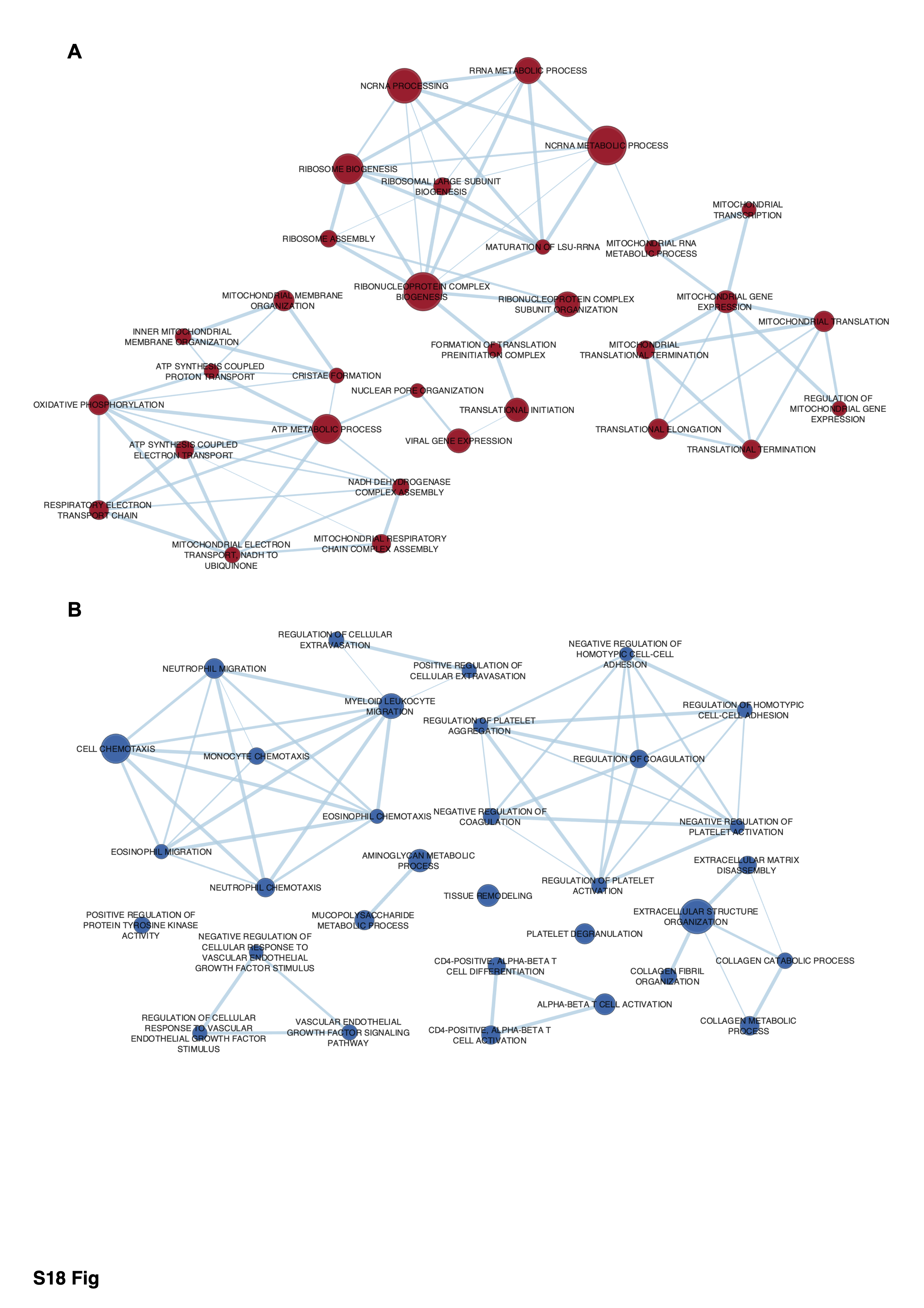

Supplement: S18 Fig — Enrichment maps for representative gene sets significantly enriched in pretreatment tumor tissues of (A) responders and (B) nonresponders with LUSC. Each node denotes a distinct gene set, and the size of the node is proportional to the number of genes in the set. The thickness of the edges (pale blue lines) represents the degree of overlap between the two connected gene sets. (TIFF) [file pone.0260500.s018.tiff]

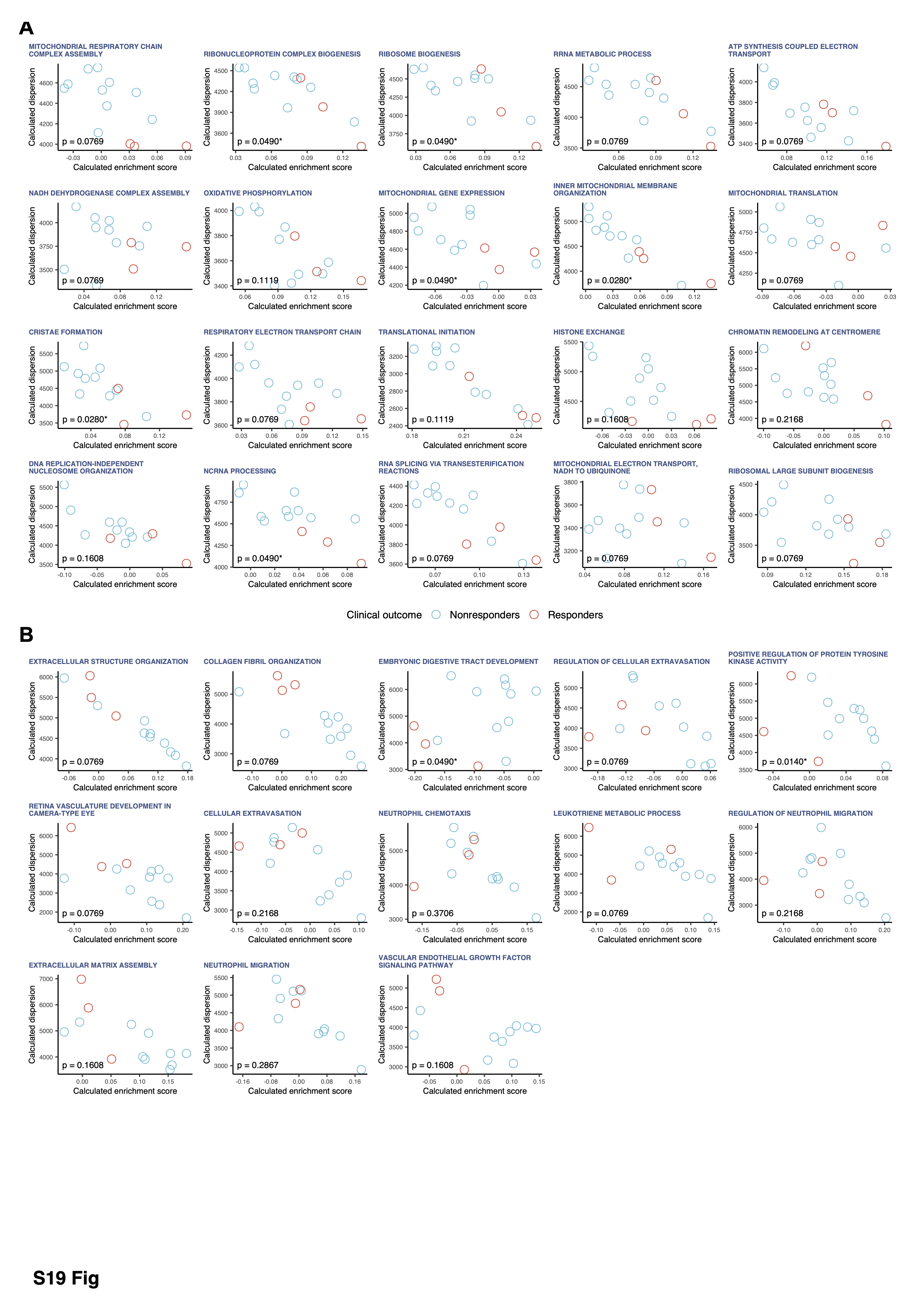

Supplement: S19 Fig — For each gene set in the top 20 GSEA gene sets that were enriched in pretreatment tumor tissues of responders (A) and nonresponders (B) (except for those shown in Fig 5A) with LUSC, the enrichment scores and dispersions were calculated using singscore. Red circles denote responders (n = 3); cyan circles, nonresponders (n = 10). The enrichment scores were analyzed using the Wilcoxon rank sum test (*p < 0.05). (TIFF) [file pone.0260500.s019.tiff]

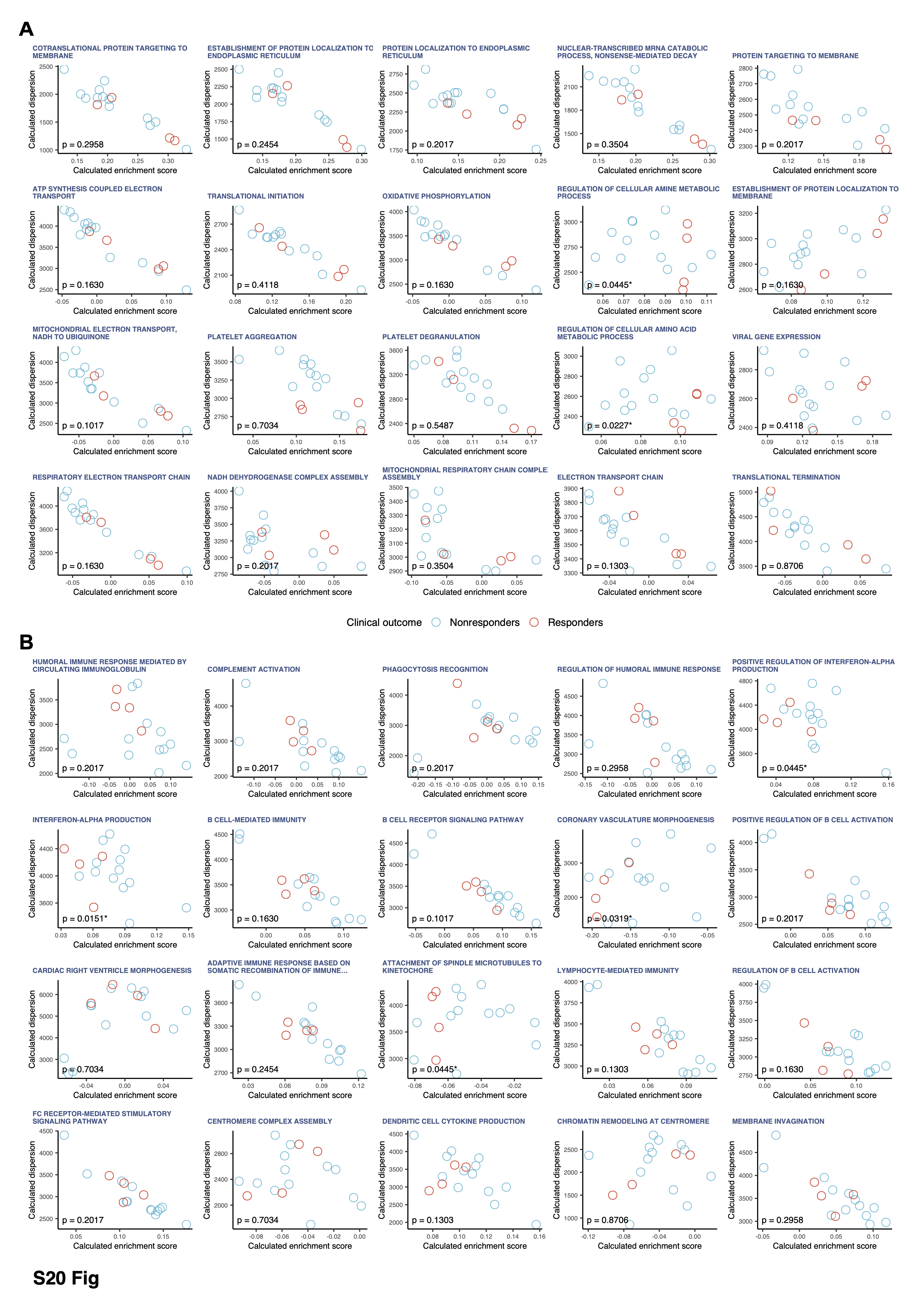

Supplement: S20 Fig — For each gene set in the top 20 GSEA gene sets that were enriched in pretreatment WB of responders (A) and nonresponders (B) with LUSC, the enrichment scores and dispersions were calculated using singscore. Red circles denote responders (n = 4); cyan circles, nonresponders (n = 13). The enrichment scores were analyzed using the Wilcoxon rank sum test (*p < 0.05). (TIFF) [file pone.0260500.s020.tiff]

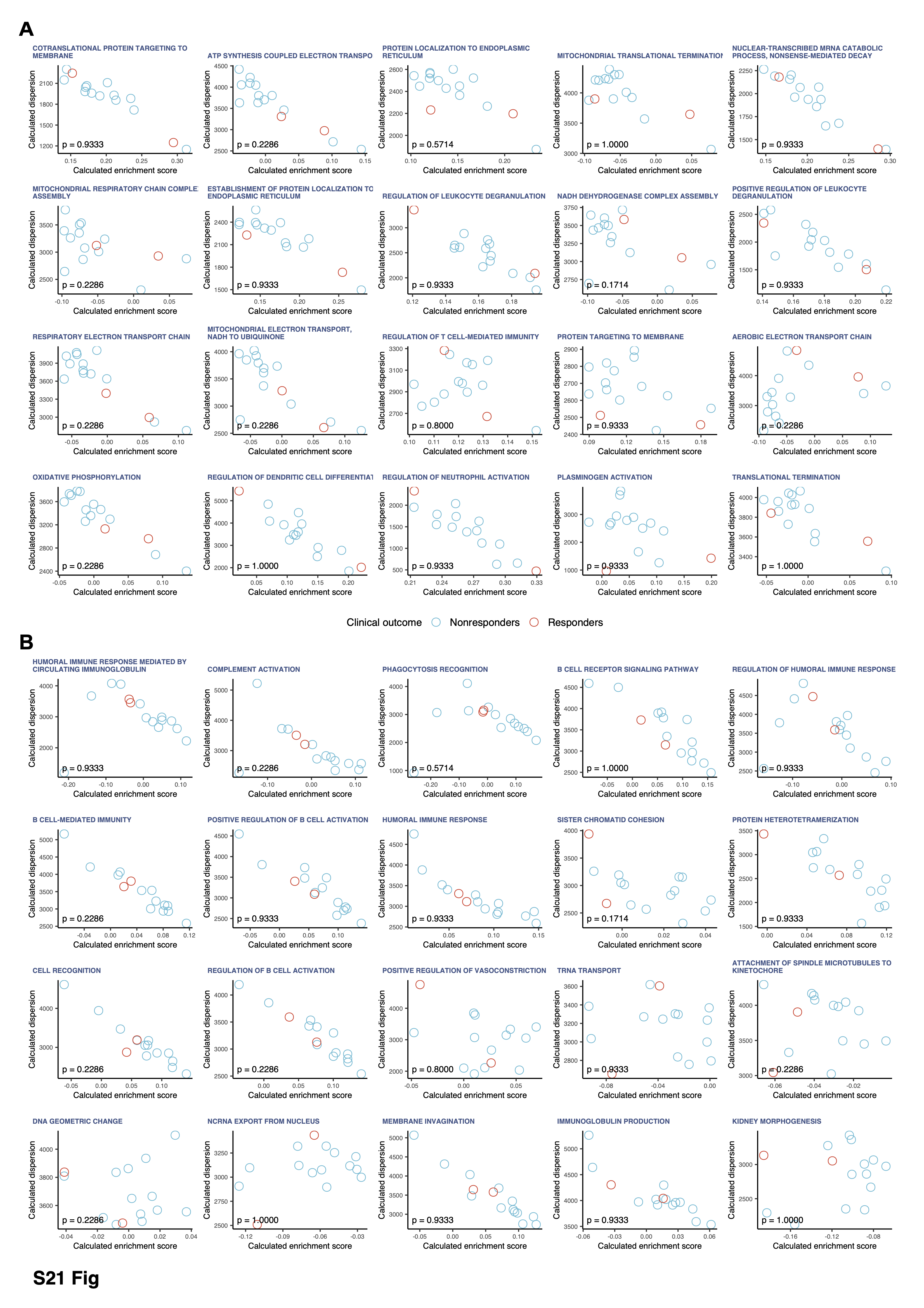

Supplement: S21 Fig — For each gene set in the top 20 GSEA gene sets that were enriched in on-treatment WB of responders (A) and nonresponders (B) with LUSC, the enrichment scores and dispersions were calculated using singscore. Red circles denote responders (n = 2); cyan circles, nonresponders (n = 13). The enrichment scores were analyzed using the Wilcoxon rank sum test. (TIFF) [file pone.0260500.s021.tiff]

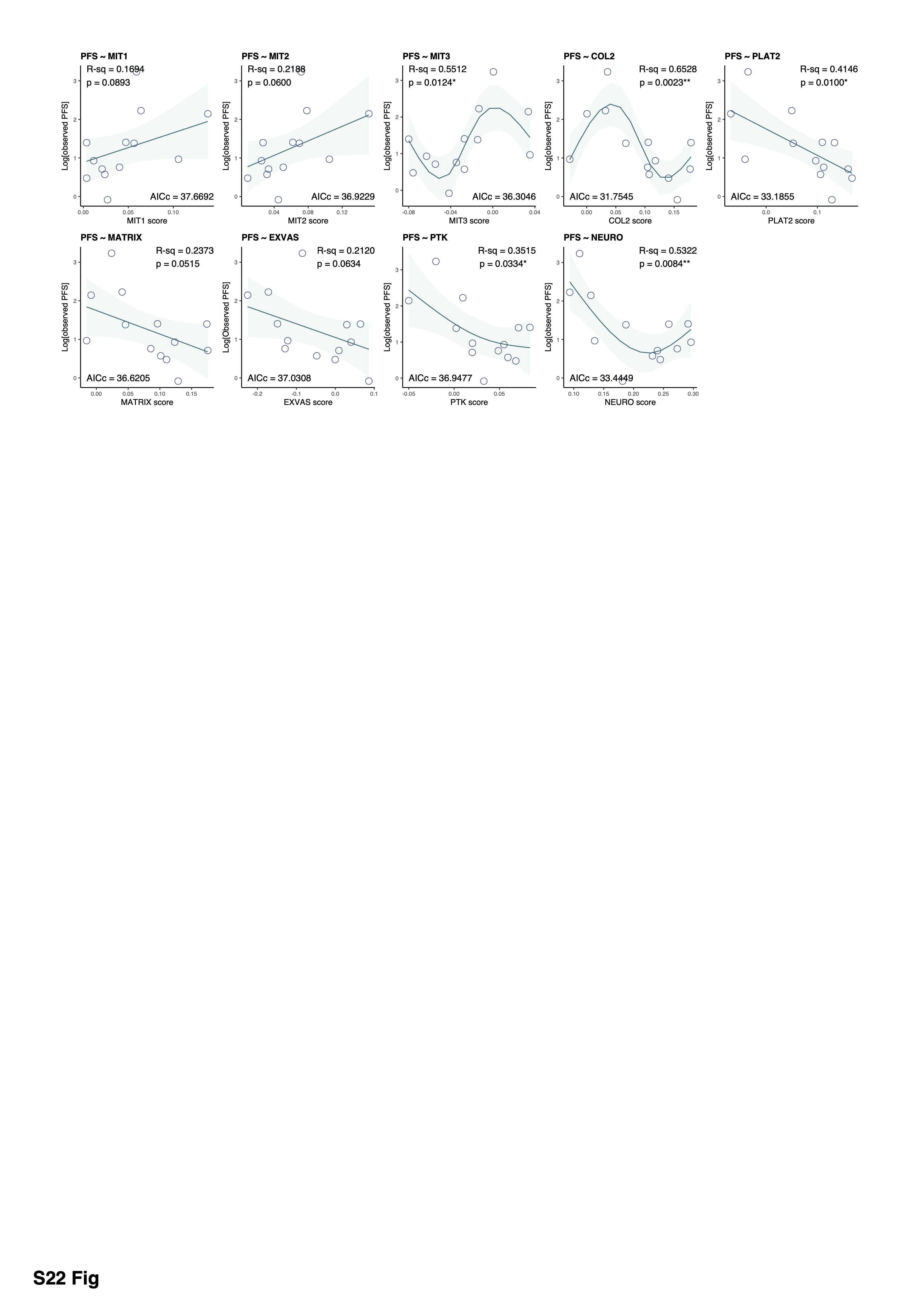

Supplement: S22 Fig — The fitted line on each scatter plot represents the regression model using a cubic spline and 95% confidence interval. The accuracy of the fit was assessed by calculating the adjusted R-squared (R-sq) and p-values (*p < 0.05 and **p < 0.01). (TIFF) [file pone.0260500.s022.tiff]

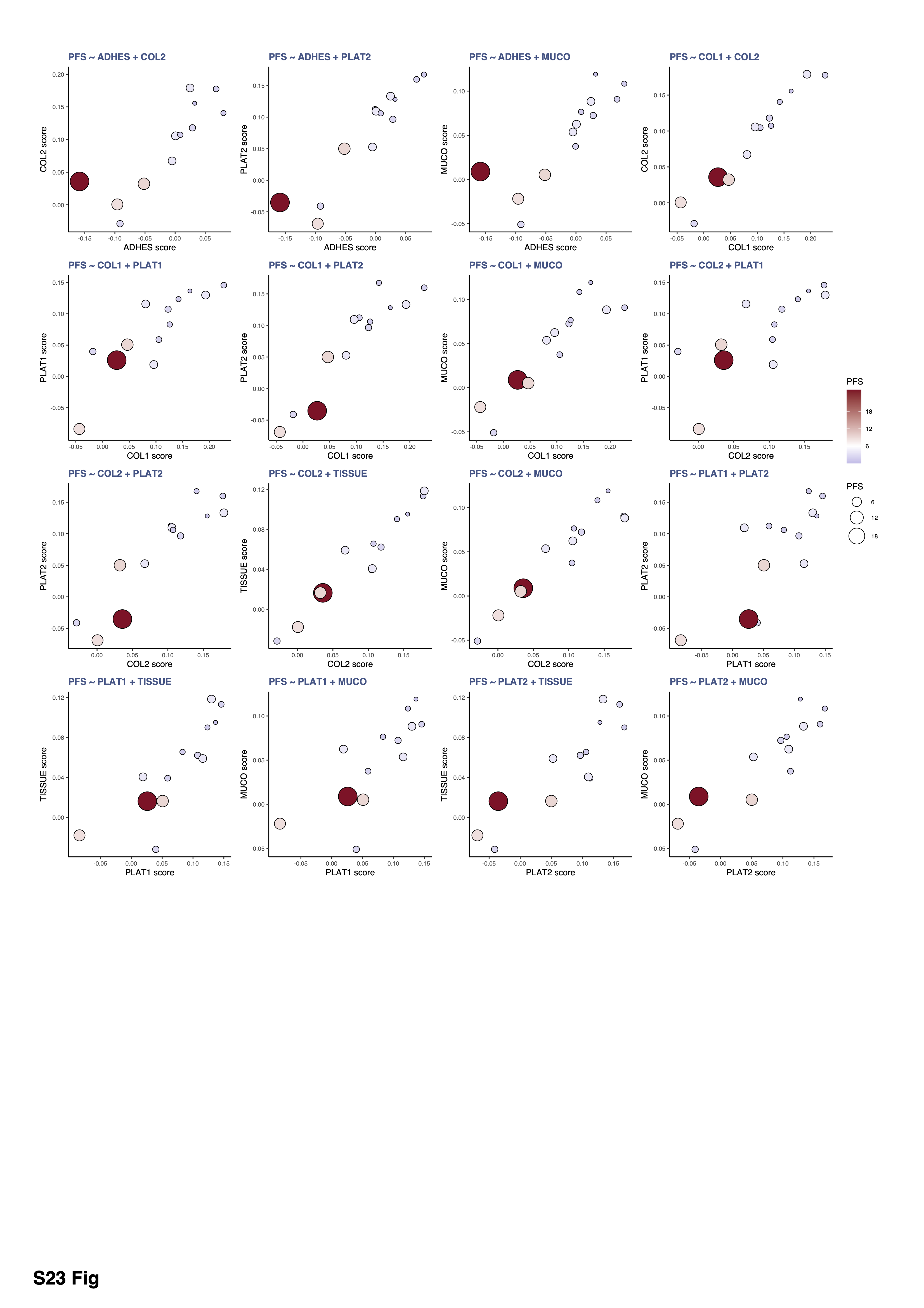

Supplement: S23 Fig — Each bubble represents a patient, and the size of the bubble is proportional to the PFS time. On a gradient color scale based on the PFS time, bubbles representing responders were assigned colors ranging from white to dark red; nonresponders, ranging from white to lavender. (TIFF) [file pone.0260500.s023.tiff]

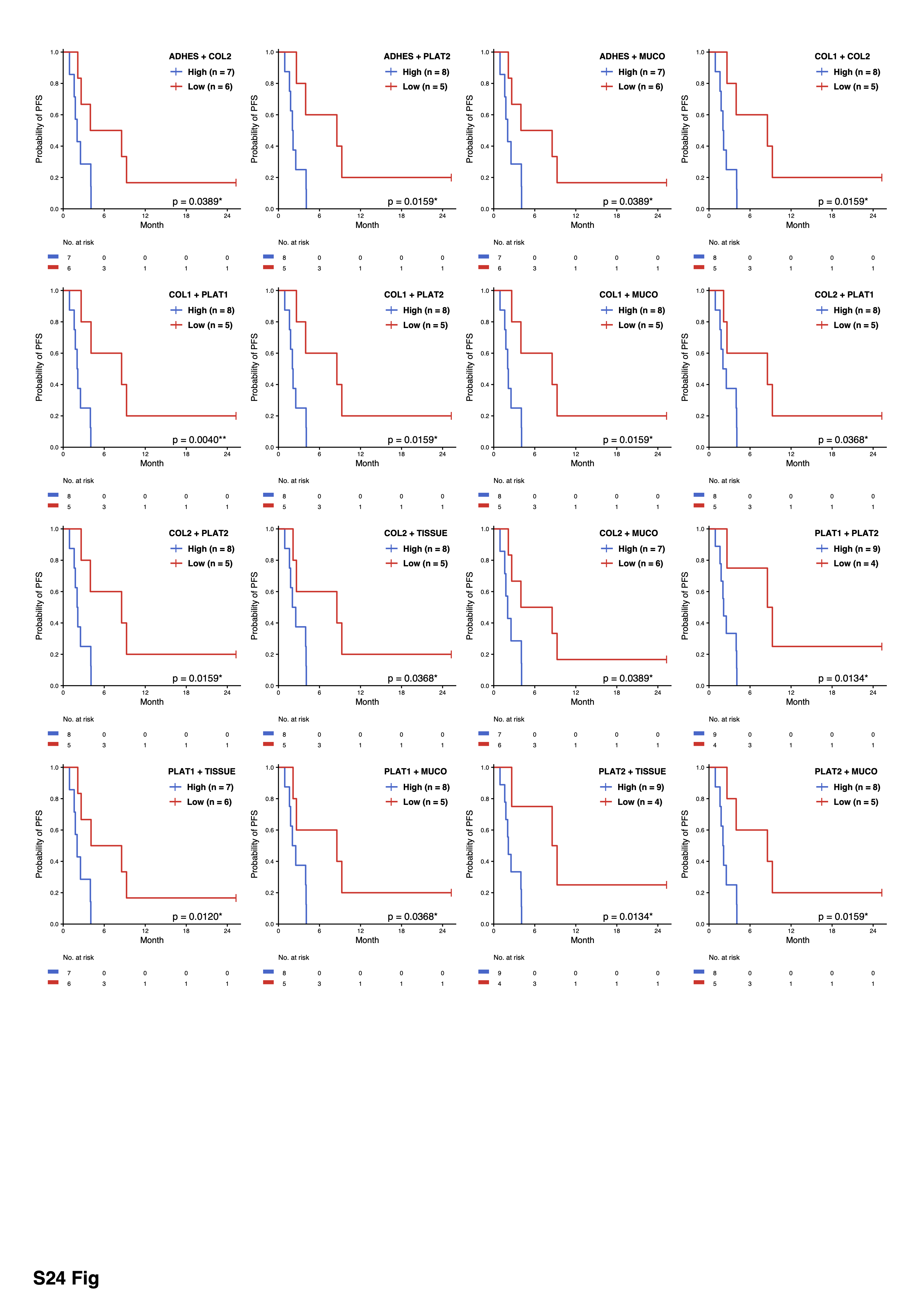

Supplement: S24 Fig — LUSC patients with both scores below the median are defined as ‘Low’; the others, as ‘High’. The p-values were calculated by the two-sided log-rank test (*p < 0.05 and **p < 0.01). (TIFF) [file pone.0260500.s024.tiff]

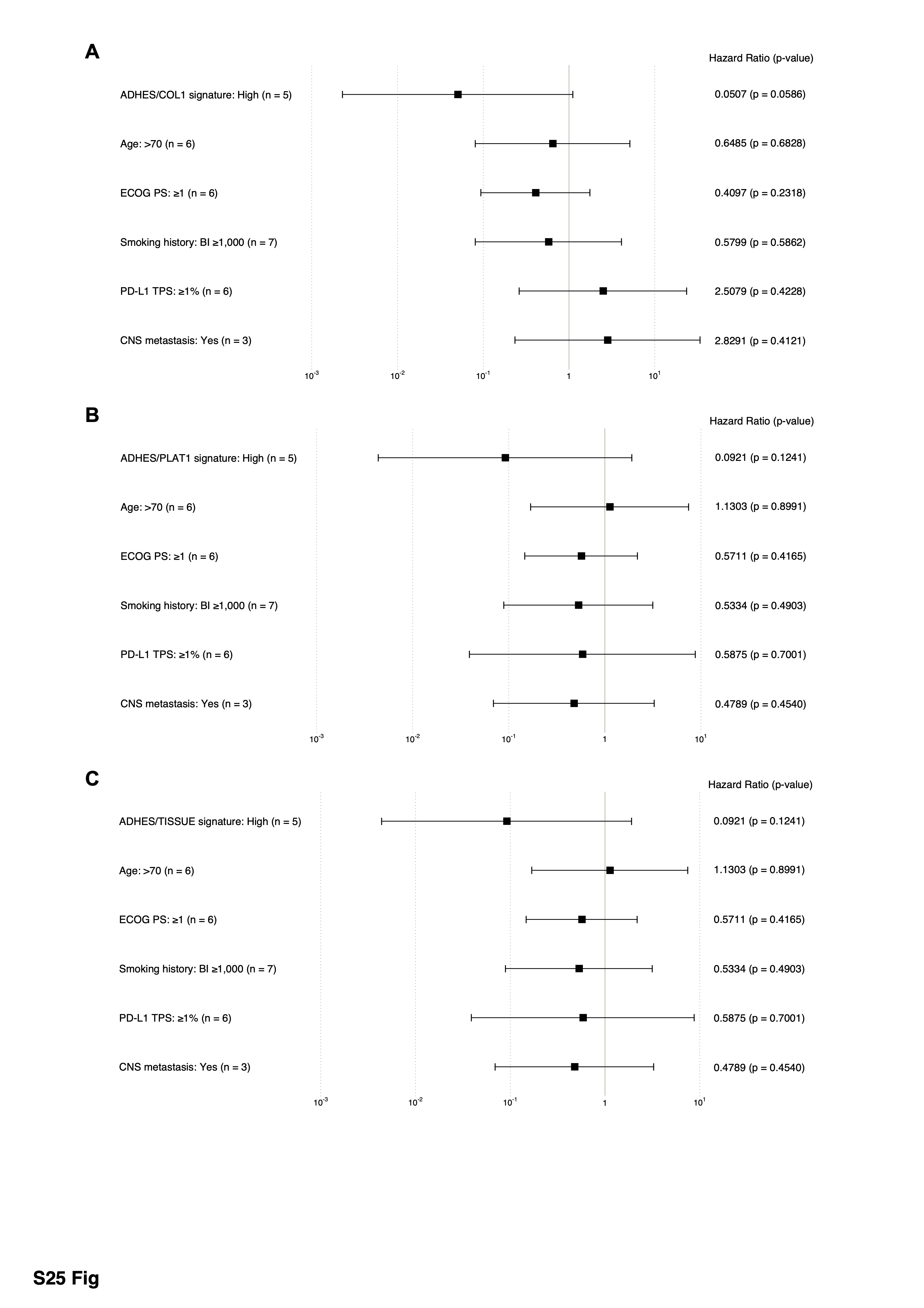

Supplement: S25 Fig — Squares represent estimated hazard ratios and whiskers represent the 95% confidence intervals. Hazard ratios less than 1 indicate improved PFS time. (TIFF) [file pone.0260500.s025.tiff]

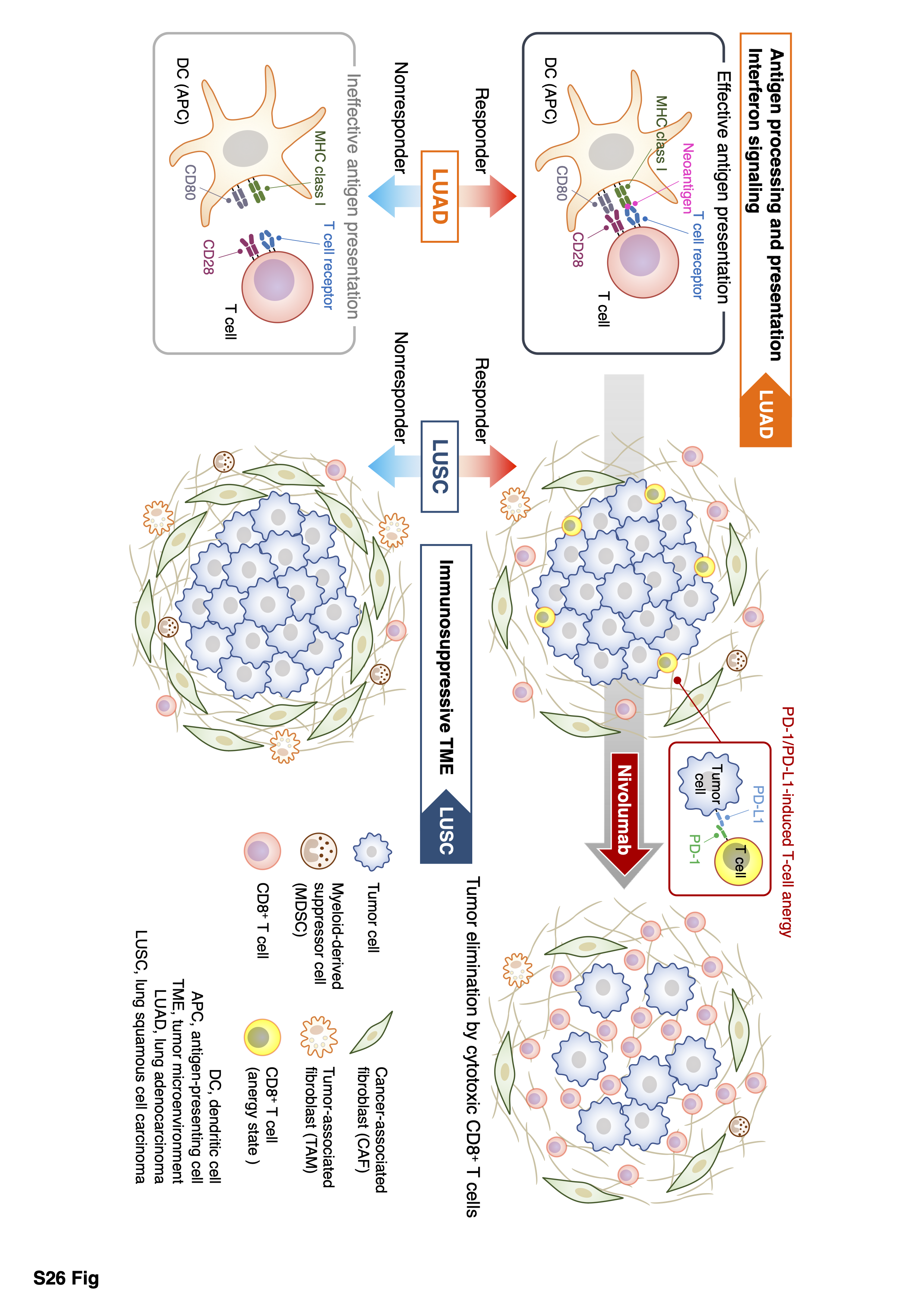

Supplement: S26 Fig — The success of nivolumab monotherapy depends on the inherent immunogenicity of the tumor itself in LUAD and the preexisting TME favoring an antitumor immune response in LUSC. (TIFF) [file pone.0260500.s026.tiff]
