# Supplementary material for: Gene expression signatures as candidate biomarkers of response to PD-1 blockade in non-small cell lung cancers
Source: PLoS One. 2021 Nov 29;16(11):e0260500. doi: 10.1371/journal.pone.0260500 (PMC8629226; doi:10.1371/journal.pone.0260500)
Supplement: S1 Table — (DOCX) [file pone.0260500.s027.docx]

**S1 Table. Assessment of tumor response to nivolumab monotherapy according to RECIST v1.1.**

| Total patients (n = 40) | | no. (%) |
| --- | --- | --- |
| Overall objective response | | 8 (20.0) |
| Best overall response | Complete response | 0 (0.0) |
|  | Partial response | 8 (20.0) |
|  | Stable disease | 16 (40.0) |
|  | Progressive disease | 14 (35.0) |
|  | Not determined | 2 (5.0) |
| Duration of response | Median - month | 3.6 |
|  | Range - month | 0.5–26.7+ |
